# Molecular Epidemiology and Phylogenetic Analysis of Peste des Petits Ruminants Virus Circulating in Sheep in Bangladesh

**DOI:** 10.1155/2023/1175689

**Published:** 2023-03-14

**Authors:** Mohammad Mojibur Rahman, Md. Saiful Islam, Abdullah Al Momen Sabuj, Md. Golzar Hossain, Md. Alimul Islam, Jahangir Alam, Md. Ershaduzzaman, Sukumar Saha

**Affiliations:** ^1^Department of Microbiology and Hygiene, Faculty of Veterinary Science, Bangladesh Agricultural University, Mymensingh 2202, Bangladesh; ^2^Bangladesh Civil Service Livestock Academy, Savar, Dhaka 1349, Bangladesh; ^3^Animal Biotechnology Division, National Institute of Biotechnology, Ganakbari, Ashulia, Savar, Dhaka 1349, Bangladesh; ^4^Krishi Gobeshona Foundation, Bangladesh Agricultural Research Council, Farmgate, Dhaka 1215, Bangladesh

## Abstract

Peste des petits ruminants (PPR) is a viral disease of small ruminants that is highly contagious, severe, reportable, and economically important. The present study was conducted to detect the PPR virus (PPRV) circulating in sheep in Bangladesh to determine its association with epidemiological risk factors and the degree of relationship between the F and H genes of the PPRV of sheep with those of other sheep and goat isolates. A cross-sectional study was conducted in five selected districts of Bangladesh to collect data on locations, ecological zones, breeds, age, sex, sources, time period, and farming systems using a structured questionnaire accompanied by face-to-face interviews. During sampling, 250 nasal swab samples were collected from live sheep with the typical clinical signs of PPR. Thereafter, a reverse-transcriptase polymerase chain reaction (RT-PCR) assay was employed to detect PPRV using the F and H genes. Risk factors were determined using bivariable and multivariable logistic regression analyses. Phylogenetic analysis of the detected PPRV was performed using MEGA software after sequencing both F and H genes. Using RT-PCR, 35.6% (89/250, 95% CI: 29.6%–41.6%) of the samples were found to be positive for PPRV. Locations, breeds, sources, and feeding systems were identified as potential molecular epidemiological risk factors for PPRV infection in a multivariate logistic regression model. Nucleotide sequencing and phylogenetic analysis showed that the PPRV strain was genetically related to the lineage IV virus isolates. For the F gene, the sequence divergence of our gene and other selected genes ranged from 0.01% to 0.018% within lineage IV, and the similarity ranged from 98.2% to 99.0%. In the case of the H gene, similar results were also observed in divergence, ranging from 0.017% to 0.083% among lineage IV and others, and similarity varied from 91.7% to 98.3%. To the best of our knowledge, this is the first study in Bangladesh conducted to determine the RT-PCR-based molecular epidemiology of PPRV in sheep. This study highlights the importance of establishing successful interventions for managing PPRV infections in small ruminants in Bangladesh.

## 1. Introduction

Peste des petits ruminants (PPR) virus (PPRV), a member of the genus *Morbillivirus* in the family *Paramyxoviridae*, is responsible for an acute, highly communicable, and generally deadly disease known as PPR in sheep and goats [1]. PPR is one of the major limiting factors in increasing the productivity of small ruminants in developing nations, and it has a disproportionately negative impact on the income of impoverished farmers [2]. The Food and Agriculture Organization (FAO) and the World Organization for Animal Health (WOAH) set the goal of eradicating PPR by 2030, following the successful eradication of rinderpest [3]. After being ignored for a long time, it is now being detected in the majority of countries in Asia, Africa, and the Near and Middle East, causing significant losses to livestock.

PPRV has an enveloped, single-stranded negative-sense RNA genome that is 15,948 nucleotides long. PPRV genome encodes six structural proteins, which are listed as follows: the nucleocapsid (N) protein, the phosphoprotein (P), the large polymerase (L) protein, the matrix (M) protein, the fusion (F) protein, and the hemagglutinin (H) protein. Two more nonstructural proteins (C and V) are also found [4, 5]. Gene sequence analysis has aided in the classification of PPRV strains into four lineages (I, II, II, and IV) found in different parts of the world. Western and Central Africa have detected PPRV isolates of lineages I and II, whereas eastern Africa and Asia (including the southern section of the Middle East) are hotspots for lineages III and IV, respectively [6–9].

The severity of PPR varies from peracute to acute to subacute to subclinical, depending on the variety of risk factors (e.g., age, sex, breed, sources, farming period, housing, and feeding systems) and the pathogenicity of the virus. The acute form of PPR is the most prevalent type and is characterized by high fever, ocular and nasal discharge, pneumonia, rapid depression, severe diarrhea, anorexia, and mouth erosive lesions [5, 10]. The disease is most prevalent in third world countries, including Bangladesh, and more specifically in regions where the farming of small ruminants is a significant contributor to both global trade and food production. Moreover, although a highly effective vaccine is available, PPR has continued to spread worldwide. It is possible that diseases could spread to PPR-free nations via unlawful transport of contaminated animal products [11].

PPR is now widespread and poses a serious danger to the growth of the sheep and goat farming industries in Bangladesh. Several studies have detected PPRV in goats in Bangladesh using molecular approaches [12–17]. Although PPR has been prevalent in Bangladesh for almost two decades, the epidemiological association and spread of PPRV in the sheep population have not yet been well elucidated. In addition, PPR persists in small ruminants in Bangladesh, necessitating molecular characterization of the spreading viral strains and additional phylogenetic analyses. Previously, Rahman et al. [18] reported PPR infection in only one sheep in Bangladesh by a reverse-transcriptase polymerase chain reaction (RT-PCR) assay; however, they did not focus on the molecular epidemiology of PPRV in the sheep population in Bangladesh. To the best of our knowledge, there are no reports on RT-PCR-based epidemiology of PPRV in sheep in Bangladesh. Therefore, the present study was conducted to detect PPRV using a molecular approach, investigate their association with various epidemiological traits, and characterize the phylogenetics of PPRV isolated from sheep in Bangladesh.

## 2. Materials and Methods

### 2.1. Ethical Statement

The Animal Welfare and Ethics Committee of Bangladesh Agricultural University in Mymensingh, Bangladesh (AWEEC/BAU/2017(17)), approved the study procedures. The verbal consent of sheep farmers was acquired before the collection of any sample. Two expert veterinarians, two microbiologists, and one enumerator were involved in the sample and data collection.

### 2.2. Study Sites

**Figure 1:**
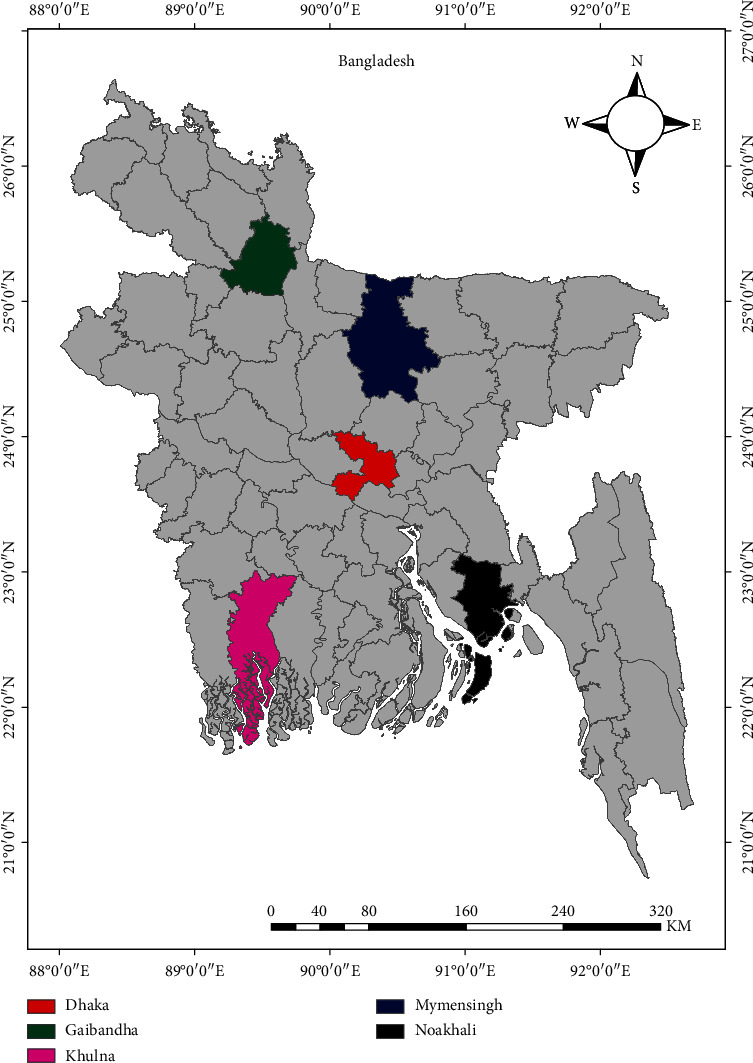
The map of the sampling sites during the present study. The map was prepared with ArcMap.v.10.7 (ArcGIS Enterprise, ESRI, Redlands, CA, USA).

From October 2014 to March 2017, this study was conducted in three ecological zones: plane land (Mymensingh (24.7471°N and 90.4203°E) and Dhaka (23.8105°N, 90.3372°E) districts), coastal region (Noakhali (22.8246°N and 91.1017°E), and Khulna (22.8456°N and 89.5403°E) districts), and the Jamuna River basin (Gaibandha (25.3290°N and 89.5415°E)) (Figure 1). The aforementioned study sites were selected based on factors, including geography, ecology, the presence of more sheep than average, husbandry methods, and the volume of animals moving through the areas.

### 2.3. Epidemiological Data Collection

The Regional Diagnostic Laboratory for PPR, Bangladesh Livestock Research Institute, Savar, Dhaka, generated the disease report form that was used in this study. It included general data, such as the name and address of farmers, farm site and size, morbidity and mortality, age, sex, breed of affected sheep, hygienic conditions of the farm, climatic conditions during outbreaks, and clinical information. During the data collection, we selected nine independent variables as possible risk factors for RT-PCR-positive PPRV infection in sheep: (1) district sampling; (2) ecological zones; (3) crossbreeding (crossed between native and Perendale sheep, native and Dorper sheep, or Chottanagpuri crossbred) or native; (4) male or female sheep; (5) age variations; (6) whether sheep were born in the farm/house or brought from markets or imported from neighboring countries; (7) season sampling; (8) variations in rearing systems; and (9) differences in feeding systems. Face-to-face interviews with farm owners were conducted by expert veterinarians (*n* = 2), microbiologists (*n* = 2), and a trained enumerator (*n* = 1). The questionnaire was written in English; however, the enumerator translated it into a local language (Bengali) and delivered it to farm owners during data collection. The report forms were filled out during the visit to each sheep farm.

### 2.4. Sample Collection and Processing

Nasal swab samples were collected aseptically from live sheep with typical clinical signs such as high fever, nasal and oral discharge, ulcers and nodules in the oral cavity, respiratory issues, abortions, and even mortality. Swab samples were collected using sterile swab sticks and stored in a viral transport medium. The samples were maintained on ice during collection and transport to the laboratory. Swab samples were processed following the guidelines described by Wasee Ullah et al. [19]. Briefly, the cotton portion of the swab was carefully detached from the swab stick using sterile forceps and scissors. The swab was then placed in a sterile Eppendorf tube comprised of 1.5 mL of sterile phosphate-buffered saline. The swab was thoroughly pressed in a 1.5 mL Eppendorf tube and spun at 9,200 × *g* for 3–5 min at 4°C. The supernatant was collected and stored at −70°C until further analysis.

### 2.5. Molecular Detection of PPRV

The samples were subjected to a RT-PCR to determine the presence of PPRV RNA [20]. Viral RNA was extracted from the processed nasal swab samples using the EZ-10 Spin Column Total RNA Mini-Prep Kit (Cat Bs1361 BIO BASIC Inc, USA) following the manufacturer's instructions. First, the RT-PCR protocol was standardized with RNA extracted from a sample using the Qiagen® RNeasy RT-PCR Mini Kit (Qiagen, Germany), as recommended by the manufacturer, with two sets of primers specific for the F gene (PPRVF1b: 5′-AGTACAAAAGATTGCTGATCACAGT-3′; PPRVF2d: 5′-GGGTCTCGAAGGCTAGGCCCGAATA-3′) and the H gene (Pprh_fr1: 5′-TGTCATGTTCTTATAGAGTT-3′; Pprh_fr2: 5′-GACTGGATTACATGTTACCT-3′). Within the same tube, reverse transcription and PCR were performed in sequential order. A reverse transcriptase enzyme was used to transform the RNA collected into cDNA. The cDNA was amplified using PPRV-specific primers for the F and H genes, as previously described [21, 22].

Amplification was performed using a final reaction volume of 25 *μ*L, which contained 15 *μ*L of the master mixture (Promega, Madison, WI, USA) (5 *μ*L of 5X QIAGEN one-step RT-PCR buffer, 1 *μ*L of dNTP mix (10 mM each), 1 *μ*L of each primer, 6 *μ*L of RNAse-free water, and 1 *μ*L of QIAGEN one-step RT-PCR enzyme mix) and 10 *μ*L of template RNA. This mixture was subjected to a thermal cycling profile of initial reverse transcription (cDNA synthesis) at 50°C for 30 min and PCR activation at 95°C for 15 min. The following steps were subsequently performed: (a) for the F gene: 35 cycles of denaturation at 94°C for 1 min, annealing at 50°C for 1 min, extension at 72°C for 2 min, and final extension at 72°C for 7 min; and (b) for the H gene: 30 cycles of denaturation at 94°C for 30 s, annealing at 55°C for 30 s, extension at 72°C for 30 s, and final extension at 72°C for 7 min. Gel electrophoresis at 120 v/80 mA for 60 min on a 1.5% agarose gel in Tris-borate-EDTA buffer was used to analyze each amplified PCR product. After the gel was stained with ethidium bromide, an ultraviolet transilluminator (Biometra, Germany) was used to view the DNA bands, and the images were saved on a computer.

### 2.6. Nucleotide Sequencing of the F and H Genes of PPRV

A positive isolate from Savar, Dhaka, was selected and purified using the Wizard®SV Gel and PCR Clean-Up System (Promega, San Luis Obispo, CA) according to the manufacturer's instructions. Subsequently, the purified PPRV PCR amplicon was sent to the National Institute of Biotechnology (NIB), Savar, Dhaka, for sequencing (F and H genes of PPRV).

### 2.7. Phylogenetic Analysis

The nucleic acid sequences obtained from the PCR products of the F and H genes were aligned with other related sequences retrieved from the GenBank database. A total of 22 partial *F* gene and 25 partial *H* gene sequences of PPRV were retrieved from the NCBI GenBank online database (Supplementary Tables S1 and S2). The aligned sequences were analyzed for their evolutionary relationships using a maximum-likelihood (ML) phylogenetic tree constructed using a gamma-distributed Tamura–Nei model [23]. A total of 1000 replicates were used to determine the confidence intervals generated by the bootstrap method. ClustalW from the Geneious program was used for the alignment of the gene sequences, and MEGA 11 [24] was used to perform maximum likelihood analysis and create the tree.

### 2.8. Statistical Analysis

The combined data from the field and laboratory were incorporated into the Excel-365 (Microsoft/Office 365, Redmond, DC, USA) spreadsheet for detailed analysis of errors and discrepancies before sorting, coding, and testing to ensure their integrity. Finally, the data were exported to the Statistical Package for Social Sciences (IBM SPSS 25.0, Chicago, IL, USA) for further statistical analyses.

#### 2.8.1. Descriptive Analysis

Descriptive analysis was performed to enumerate the frequency distribution of selected epidemiological factors and the prevalence of RT-PCR-positive PPRV based on those factors. A Chi-square test for relatedness (*Z*-test for proportion) was performed to determine whether there was a statistically significant difference in the prevalence of distinct exposure factors. The statistical *p* value was set at ≤0.05. In addition, a 95% confidence interval (CI) was computed to determine the estimated values for RT-PCR-positive PPRV in sheep according to different epidemiological factors.

#### 2.8.2. Risk Factor Analysis

A bivariable logistic regression was conducted to ascertain any cross-associations between each independent variable and PPRV infection (RT-PCR positivity). Any independent variable with a *p* value ≤0.2 (*p* ≤ 0.2) in the bivariable analysis was included in the multiple logistic regression analysis.

A forward stepwise multiple logistic regression model was constructed to fit this model. The Spearman correlation matrix (by bivariate analysis) of all candidate independent variables was plotted to evaluate multicollinearity in the regression model. The Hosmer–Lemeshow test was also performed to assess the goodness-of-fit of the model, and the postestimate of the predictive ability was calculated using the receiver operating characteristic curve to check the sensitivity of the results. Finally, in the final model, variables with a *p* value less than or equal to 0.05 (*p* ≤ 0.05) were deemed risk factors for RT-PCR-positive PPRV infection in sheep. For each adjusted predictor variable, the findings were displayed as odds ratios (ORs), along with their *p* value and 95% confidence intervals (CIs).

## 3. Results

### 3.1. Descriptive Statistics of the Samples

A total of 250 sheep nasal swab samples were collected. All five districts constituted an equal number of samples (50 samples from each district), and both the plane and coastal zones accounted for the highest number of samples collected (40% each). The majority of the samples were collected from native sheep (58%), female sheep (60%), and sheep aged 13–24 months (40%). Moreover, sheep born in farms/houses (56%), during the winter season (36.8%), in a farming system (68%), and in a stall-feeding system (60%) constituted the highest percentage of the samples (Table 1).

### 3.2. Detection of PPR Virus from Suspected Field Samples

Out of 250 nasal swab samples, 89 (35.6%, 95% CI: 29.6%–41.6%) were found to be positive for PPRV using an RT-PCR assay (with both F and H genes). Among them, a significantly higher (*p* < 0.05) prevalence of RT-PCR-positive PPRV was recorded in the Dhaka district (54%, 27/50, 95% CI: 39.7%–68.3%), in crossbreed sheep (51.4%, 54/105, 95% CI: 41.7%-61–2%), male sheep (44%, 44/100, 95% CI: 34.1%–53.9%), imported sheep (76%, 19/25, 95% CI: 58.0%–93.9%), sheep that were reared in the winter season (45.7%, 42/92, 95% CI: 35.3%–56.0%), in a nonfarming system (46.3%, 37/80, 95% CI: 35.1%–57.4%), and in a grazing feeding system (46%, 46/100, 95% CI: 36.1%–55.9%) (Table 1). No significant association was observed between the PPRV detection rate in the ecological zone and the age group (Table 1).

### 3.3. Risk Factors of RT-PCR-Positive PPRV Infection in Sheep

#### 3.3.1. Bivariable Analysis

In the bivariable analysis, among the nine independent variables, only one variable (age groups of sheep) was excluded because of a *p* value higher than 0.2 (Table 2). Eight variables associated with PPRV infection, including location, ecological zone, breed, sex, sources of sheep, seasonal variations, rearing system, and feeding system, were considered as candidate variables for the multivariable logistic regression analysis (Table 2).

#### 3.3.2. Multivariable Analysis

In the multiple logistic regression model, out of eight candidate independent variables (with a *p* value ≤0.2), four variables were identified as potential risk factors for PPRV infection (Table 3). The most important risk factor was the source of sheep; imported sheep had 10 times the odds (OR = 10, 95% CI: 3.3%–30.1%, *p* < 0.001) of the prevalence of PPRV infection than sheep born in farms/houses and brought from markets. Other risk factors were location, sheep breed, and feeding system (Table 3).

### 3.4. Phylogenetic Analysis

#### 3.4.1. Construction of a Phylogenetic Tree Using Nucleotide Sequences of F and H Genes of PPR Virus Isolate and Other Globally Found PPRV Isolates

**Figure 2:**
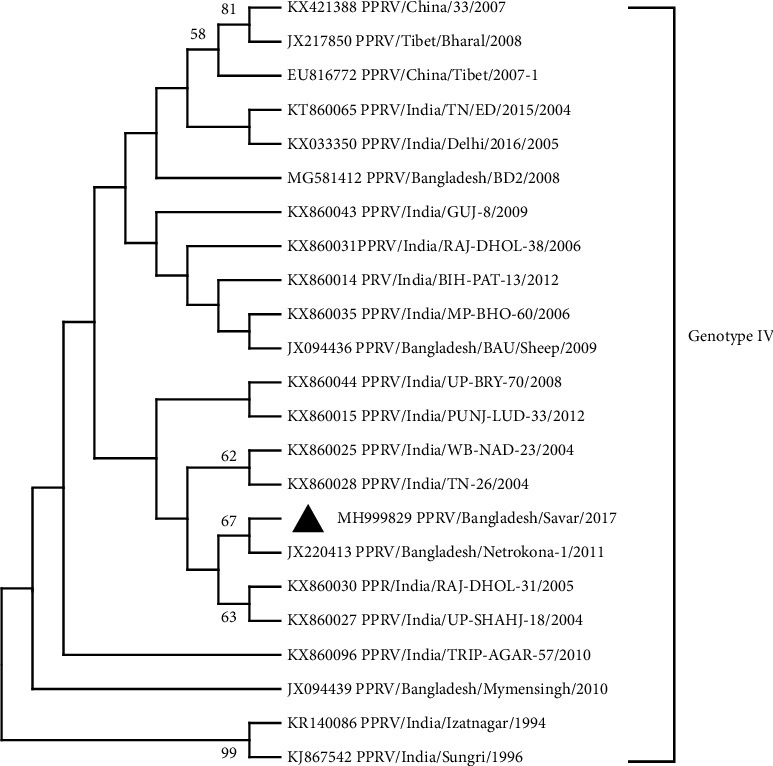
Evolutionary relationships of the partial fusion gene sequence (397 bp) of PPR viruses. The evolutionary history was inferred using the neighbor-joining method. The evolutionary distances, showed at each node, were computed using the maximum composite likelihood method and are in the units of the number of base substitutions per site. This analysis involved 23 nucleotide sequences. Evolutionary analyses were conducted in MEGA-11. Our virus is marked with a black colored triangle. Lineage of PPRV is shown at the right side of the tree.

**Figure 3:**
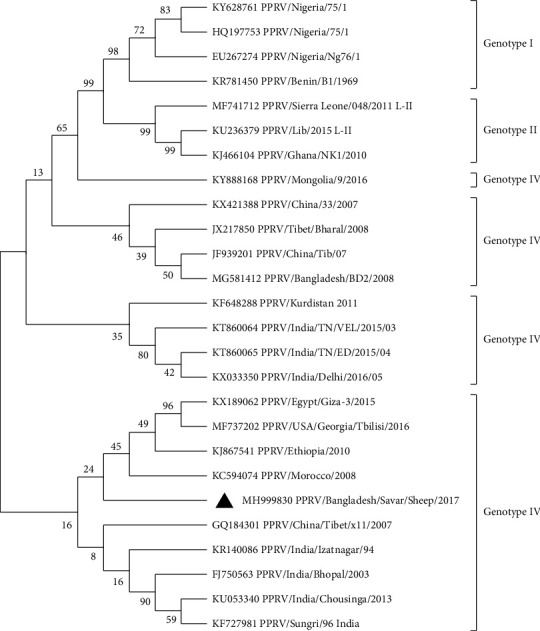
Evolutionary relationships of the partial haemagglutinin gene sequence (297 bp) of PPR viruses. The evolutionary history was inferred using the neighbor-joining method. The evolutionary distances, showed at each node, were computed using the maximum composite likelihood method and are in the units of the number of base substitutions per site. This analysis involved 26 nucleotide sequences. Evolutionary analyses were conducted in MEGA-11. Our virus is marked with a black colored triangle. Lineages of PPRV are shown at the right side of the tree.

Two phylogenetic trees (for each F gene and H gene) were constructed by aligning the nucleotide sequences acquired in this study with the sequences of other PPRV strains found in GenBank (Figures 2 and 3). The nucleotide sequence obtained was submitted to the GenBank database (accession number: MH999829 for the F gene and MH999830 for the H gene). The phylogeny based on the F gene showed that the present isolate, PPRV/BD/Savar/2017, was similar to the BD/PPR/Netrokona-1/2011 isolate from Bangladesh and PPRV/RAJ-DHOL-31/2005 and PPRVUP-SHAHJ-18/2004 isolates from India and formed a subcluster. The H gene sequence of the Bangladeshi isolate was similar to that of PPRV/Tibet/2010 and PPRV/China/33/07 isolates from China and PPRV/Izatnagar/9 and PPRV/Bhopal/03 isolates from India. The Bangladeshi isolate PPR/BD/Savar/Sheep/2017 was grouped into line IV in both cases.

#### 3.4.2. Degree of Relationship of F and H Genes of PPRV of Sheep

Within lineage IV, for the F gene, the sequence divergences of our gene with other selected F gene sequences ranged from 0.01% to 0.018%, and similarity varied from 98.2% to 99.0%. In the case of the H gene, similar results were also observed in divergence ranging from 0.017% to 0.083% among lineage IV and others, and similarity varied from 91.7% to 98.3% (Supplementary Figure S1).

## 4. Discussion

Owing to the high morbidity and mortality rates attributed to PPRV, which can range from 90% to 100% among infected small ruminants, this virus has a significant impact on the economics of the livestock industry, particularly in endemic regions such as Bangladesh [25]. Only nasal swab samples were selected for molecular detection of PPRV in this study because nasal samples are more likely to contain the virus. Nasal secretions develop alongside other often-noticed clinical signs and continue even after the temperature decreases. In addition, nasal swabs show consistent results because the virus is likely excreted for a long time by nasal excretion. Moreover, Parida et al. [26] demonstrated that nasal swab samples showed superior results to other selected samples for the detection of PPRV nucleic acids and that nasal swabs might be preferred for the detection of PPRV during an outbreak investigation. In this study, we found that PPRV was detected by F gene- and H gene-based RT-PCR in 35.6% (89/250) of nasal swab samples of sheep, which indicates the circulation of PPRV in the selected areas. Nonetheless, because only animals with clinical signs of PPR were selected for sample collection, this may not be a reliable indicator of PPRV prevalence. Several studies have detected PPRV in sheep in Bangladesh [27–29]; however, none have focused on a molecular approach. Rahman et al. [18] reported PPRV infection in only one occasional sheep sample in Bangladesh, but they did not focus on the molecular epidemiology of PPRV infection in sheep. On the contrary, in our study, for the first time, we investigated the molecular epidemiology of PPR in sheep in different districts of Bangladesh. Compared to other studies using RT-PCR assays in different countries, the prevalence of PPRV detected in the present study was lower than that reported by Yilmaz [30] (56.5%) and Mohammed et al. [11] (54.2%) but higher than that reported by Saritha et al. [31] (25%) and Altan et al. [32] (10.4%). The discrepancy could be attributed to differences in geographical location, sample type and size, farming methods, hygiene conditions, physiological health status, movement of cross-border animals, and other factors. In most cases, sheep and goats are reared together in Bangladesh for breeding and meat production. The presence of PPRV in sheep in Bangladesh suggests the potential transmission of PPRV in goat populations and indicates a significant negative impact on small ruminant production.

This study revealed that location variation is a potential risk factor for PPRV infection. Among all the locations, the Dhaka district demonstrated the highest prevalence of PPRV, with an odds ratio of 3.5 compared to the referenced study area. This may be explained by the high number of small ruminants found in that particular region because the International Cattle Market, which also functions as a goat and sheep market (where the majority of large and small ruminant purchases are made in Bangladesh), is located in the Dhaka district. Most nasal swab samples of crossbred sheep were collected from the Dhaka district. In addition, most imported small and large ruminants, including sheep, are addressed in the animal markets of the Dhaka district. In addition, the detection rate of PPRV in different districts indicates that PPR has been spreading across various regions of Bangladesh. Therefore, proper monitoring and surveillance of PPR are necessary to control PPRV infection throughout the country.

In the present study, the type of sheep (breed) was found to be another important risk factor for the occurrence of PPRV in Bangladesh, where crossed sheep had increased odds (OR = 2.1) compared to native sheep. Although we did not measure the antibody levels in crossbred and native or indigenous sheep, it is possible that crossbred sheep do not have a sufficient level of immunity compared to native or indigenous sheep in protecting against PPRV infection. [33]. Despite being less susceptible to the virus themselves, showing fewer clinical indications of infection, and excreting less virus, local breeds have the potential to spread PPRV to other sheep [34]. In recent years, unrestricted animal movement across international boundaries has resulted in a significant increase in the number of imported animals in Bangladesh. This may explain the higher occurrence of PPRV in crossbred sheep. Therefore, we suggest maintaining regular vaccination and surveillance programs in all sheep types to reduce the occurrence of PPRV infection in Bangladesh.

One of the most important risk factors for the occurrence of PPRV in sheep is their source (which indicates how selected sheep were introduced to the farms). Sheep that were imported from neighboring countries had increased odds of PPRV infection (OR = 10) compared to sheep from animal markets and those reared at home. According to the current investigation results, new sheep from various live-animal markets are a major source of PPRV infection and spread on farms. Although native sheep constitute the vast bulk of the flocks of Bangladesh, some farmers are more interested in breeding foreign breeds. Farmers either import sheep from other parts of the country or from neighboring countries or bring domestic sheep from other areas onto their farms without going through a quarantine period. There is some evidence that PPR is associated with the transfer of newly infected animals from markets to farms or related to close contact between healthy and sick sheep that were transported to the animal market for sale but were not sold and then returned to the farms [35]. Regular monitoring systems and appropriate regulations should be employed for the purchases of small ruminants.

Feeding systems are a potential risk factor affecting the prevalence of PPRV infection in sheep in Bangladesh. PPRV was more likely to occur in grazing systems (OR = 2.1) than in stall-feeding systems. In Bangladesh, during open grazing, sheep are mixed with sheep and goats from other farms. This environment may create a suitable atmosphere for the transmission of PPRV in sheep. Farmers often do not follow isolation and quarantine procedures to prevent infection in open grazing systems. According to a study by Bwihangane et al. [36], animals that were allowed to graze in free environments had a higher rate of PPRV infection than those maintained in stall-grazing farming systems. Because infected animals can spread diseases to healthy animals, farms that follow the grazing feeding system must be maintained under regular surveillance to control disease transmission from animal to animal or farm to farm.

Additional bivariate analysis components with substantial associations were excluded from the final model, including sex variations, variations in rearing times (seasons), and systems. The prevalence of PPRV was significantly higher in male sheep than in female sheep, which is consistent with a previous study [37]. Because of variations in their genetic makeup, male animals are believed to be more likely to contract diseases [38]. While the specific host and pathogen associated with diversity still remain unelucidated, we do know that the increasing demand for male animals for meat has driven them to the market, where they are more likely to be infected than females, which are normally maintained at home for reproductive purposes [39]. In addition, significant variation in the prevalence of PPRV infection was reported in rearing times (seasons) and rearing systems. The winter season had a significantly higher prevalence than the summer and rainy seasons. It is possible that the seasonal distribution of PPR outbreaks is also influenced by climatic conditions that are conducive to the survival and propagation of the virus [40]. The dusty and dry winds that are characteristics of the winter season of the year assist in increasing the spread of the PPR [41]. Moreover, the farming system had a lower occurrence of PPRV infection than the nonfarming system. Maintaining sheep in a nonfarming system may facilitate contact with other diseased sheep or goats, in turn, creating another route of PPR infection.

Using F and H gene analyses, we observed that Bangladeshi strain PPR/BD/Savar/Sheep/2017 belongs to lineage IV. Although both PPRV/Bangladesh/BD2/2008 and the older isolate BD/PPR/Netrokona-1/2011 were clustered in lineage IV, they belonged to separate subclusters. However, whether this occurred as a result of genetic drift or a recent introduction remains unclear. Intriguingly, phylogenetic analysis of the F gene revealed that the current isolate PPRV/BD/Savar/2017 formed a subcluster with the Bangladeshi BD/PPR/Netrokona-1/2011 isolate and the two Indian isolates PPRV/RAJ-DHOL-31/2005 and PPRV/UP-SHAHJ18/2004. The PPR/BD/Savar/Sheep/2017 isolate shared similarities in its H gene sequence with two Chinese isolates, PPRV/Tibet/2010 and PPRV/China/33/07, and two Indian isolates, PPRV/Izatnagar/9 and PPRV/Bhopal/03. These results indicate the formation of new branches within lineage IV. The sequence divergence within lineage IV ranged from 0.000% to 0.021%, and the data demonstrated that the F gene sequences were substantially more conserved. H gene sequences, in contrast, showed considerable variation, with a divergence range of 0.000%–0.087% between lineage IV and other lineages. Classifying PPRV into lineages based on the F gene sequences may provide better insights into molecular epidemiology [42, 43]. As a result, molecular characterization of circulating strains is an essential tool for understanding the epidemiology of PPRV and keeping tabs on outbreaks across the country. These data can assist in describing eventual changes in virulence across strains and establishing the variety and dispersion of variants in the field, as well as help determine the geographical source of a virus and predict the danger of its entrance into the herd. Such insights will also aid in guiding and improving current efforts to contain and eradicate this problem.

The limitation of our study is that we could not analyze the F and N genes of PPRV from a sufficient number of isolates because of funding limitations. We believe that information on the amino acid changes in the F and H genes of all PPRV isolates detected in this study would have added value to the results. Therefore, we recommend that further studies should be conducted to determine the genetic diversity of PPRV isolates in sheep in Bangladesh using whole-genome sequencing.

## 5. Conclusion

This study, for the first time, provides important information about the frequency of PPRV infection (detected by a molecular approach) in sheep and the factors that increase their susceptibility to this disease in certain regions of Bangladesh. This study confirms that PPRV infection is circulating among the sheep population in Bangladesh, indicating possible transmission from animal to animal and farm to farm. Therefore, we advocate a widespread vaccination program in conjunction with a comprehensive disease surveillance system to contain the epidemic in Bangladesh.

## Figures and Tables

**Table 1:** Prevalence of RT-PCR-positive PPRV in relation to different factors.

Variables	Categories	No. of samples collected (%)	Positive, *n*(%)	95% CI (%)	*p* value
Districts/locations	Mymensingh	50 (20)	14 (28^a^)	15.1–40.9	0.046
	Dhaka	50 (20)	27 (54^b^)	39.7–68.3	
	Noakhali	50 (20)	16 (32^a^)	18.6–45.4	
	Khulna	50 (20)	17 (34^a^)	20.4–47.6	
	Gaibandha	50 (20)	15 (30^a^)	16.8–43.2	

Ecological zones	Plane land	100 (40)	41 (41^a^)	31.2–50.8	0.325
	Coastal	100 (40)	33 (33^a^)	23.6–42.4	
	Jamuna river basin	50 (20)	15 (30^a^)	16.8–43.2	

Breed	Crossbreds	105 (42)	54 (51.4^a^)	41.7-61-2	<0.001
	Native	145 (58)	35 (24.1^b^)	17.1–31.2	

Sex	Male	100 (40)	44 (44^a^)	34.1–53.9	0.024
	Female	150 (60)	45 (30^b^)	22.6–47.4	

Age	0–6 months	40 (16)	14 (35^a^)	19.6–50.5	0.980
	7–12 months	42 (16.8)	15 (35.7^a^)	20.6–50.8	
	13–24 months	100 (40)	37 (37^a^)	27.4–46.6	
	>24 months	68 (27.2)	23 (33.8^a^)	22.3–45.4	

Sources	Animal market	85 (34)	33 (38.8^a^)	28.3–49.4	<0.001
	Imported	25 (10)	19 (76^b^)	58.0–93.9	
	Born in farm/house	140 (56)	37 (26.4^a^)	19.0–33.8	

Seasons	Winter (Nov–Feb)	92 (36.8)	42 (45.7^a^)	35.3–56.0	0.037
	Rainy (Jul–Oct)	80 (32)	25 (31.3^a,b^)	20.9–41.6	
	Summer (Mar–Jun)	78 (31.2)	22 (28.2^b^)	17.9–38.4	

Rearing systems	Nonfarming	80 (32)	37 (46.3^a^)	35.1–57.4	0.016
	Farming	170 (68)	52 (30.6^b^)	23.6–37.6	

Feeding systems	Stall feeding	150 (60)	43 (28.7^a^)	21.4–35.9	0.005
	Grazing	100 (40)	46 (46^b^)	36.1–55.9	

Values with different superscripts differ significantly (*p* < 0.05) within the variable under assessment; *n*, number of positive isolates; CI, confidence interval; Nov, November; Feb, February; Jul, July; Oct, October; Mar, March; Jun, June.

**Table 2:** Epidemiological factors associated with the prevalence of RT-PCR-positive PPRV infection in sheep in Bangladesh.

Variables^a^	Categories	Odds ratio	95% CI	*p* value
Districts/locations	Mymensingh	Ref.		
	Dhaka	3.0	1.3–6.9	0.009
	Noakhali	1.2	0.5–2.9	0.663
	Khulna	1.3	0.6–3.1	0.517
	Gaibandha	1.1	0.5–2.6	0.826

Ecological zones	Plane land	1.6	0.8–3.4	0.191
	Coastal	1.2	0.6–2.4	0.711
	Jamuna river basin	Ref.		

Breed	Crossbreds	3.3	1.9–5.7	<0.001
	Native	Ref.		

Sex	Male	1.8	1.1–3.1	0.024
	Female	Ref.		

Age	0–6 months	1.1	0.5–2.4	0.901
	7–12 months	1.1	0.5–2.4	0.839
	13–24 months	1.2	0.6–2.2	0.673
	>24 months	Ref.		

Sources	Animal market	1.8	0.9–3.1	0.053
	Imported	8.8	3.3–23.8	<0.001
	Born in farm/house	Ref.		

Seasons	Winter (Nov–Feb)	2.1	1.1–4.1	0.02
	Rainy (Jul–Oct)	1.2	0.6–2.3	0.676
	Summer (Mar–Jun)	Ref.		

Rearing systems	Nonfarming	1.9	1.1–3.4	0.017
	Farming	Ref.		

Feeding systems	Stall feeding	Ref.		
	Grazing	2.1	1.3–3.6	0.005

^a^Candidate variables for multiple logistic regression; CI, confidence interval; Nov, November; Feb, February; Jul, July; Oct, October; Mar, March; Jun, June.

**Table 3:** Potential risk factors of PPRV infection in sheep in multiple logistic regression.

Variables	Categories	OR	95% CI	*p* value
Districts/locations	Mymensingh	Ref.		
	Dhaka	3.5	1.4–9.1	0.008
	Noakhali	1.2	0.5–3.1	0.710
	Khulna	1.3	0.5–3.4	0.557
	Gaibandha	1.1	0.4–2.9	0.794

Breed	Crossbreds	2.1		
	Native	Ref.	1.1–4.2	0.036

Sources	Animal market	1.1	0.6–2.3	0.715
	Imported	10.0	3.3–30.1	<0.001
	Born in farm/house	Ref.		

Feeding	Stall feeding	Ref.		
	Grazing	2.3	1.2–4.5	0.015

OR, odds ratio; CI, confidence interval.

## Data Availability

The datasets used and/or analyzed during the current study are available from the corresponding author upon request.

## Supplementary Materials

Supplementary Materials Supplementary Table S1: List of accession numbers, lineages, and countries of origin of F gene isolates of PPRV in GenBank. Supplementary Table S2: List of accession numbers, lineages, and countries of origin of H gene isolates of PPRV in GenBank. Supplementary Figure S1: Similarities of our F and H gene sequences with other F (F1–F22) and H (H1–H25) gene sequences of PPR virus isolates of sheep.
