# Supplementary material for: Molecular Epidemiology and Phylogenetic Analysis of Peste des Petits Ruminants Virus Circulating in Sheep in Bangladesh
Source: Transbound Emerg Dis. 2023 Mar 14;2023:1175689. doi: 10.1155/2023/1175689 (PMC12016724; doi:10.1155/2023/1175689)
Supplement: Supplementary Materials — Supplementary Table S1: List of accession numbers, lineages, and countries of origin of F gene isolates of PPRV in GenBank. Supplementary Table S2: List of accession numbers, lineages, and countries of origin of H gene isolates of PPRV in GenBank. Supplementary Figure S1: Similarities of our F and H gene sequences with other F (F1–F22) and H (H1–H25) gene sequences of PPR virus isolates of sheep. [file 1175689.f1.docx]

**Molecular epidemiology and phylogenetic analysis of Peste des petits ruminants virus circulating in sheep in Bangladesh**

Mohammad Mojibur Rahman^1,2^, Md. Saiful Islam^1^, Abdullah Al Momen Sabuj^1^, Md. Golzar Hossain^1^, Md. Alimul Islam^1^, Jahangir Alam^3^, Md. Ershaduzzaman^4^, Sukumar Saha^1,^ *

^1^Department of Microbiology and Hygiene, Faculty of Veterinary Science, Bangladesh Agricultural University, Mymensingh-2202, Bangladesh

^2^Bangladesh Civil Service Livestock Academy, Savar, Dhaka-1349, Bangladesh

^3^Animal Biotechnology Division, National Institute of Biotechnology, Ganakbari, Ashulia, Savar, Dhaka 1349, Bangladesh

^4^Krishi Gobeshona Foundation, Bangladesh Agricultural Research Council, Farmgate, Dhaka-1215, Bangladesh

***Corresponding author:** [sukumar.saha@bau.edu.bd](mailto:sukumar.saha@bau.edu.bd)

**Supplementary Table S1.** List of accession no., lineage and country of origin of **F gene** isolates of PPRV in GenBank with their accession number (**Accession number our F gene - MH999829**)

| **Serial no.** | **Accession no.** | **F gene isolates** | **Lineage** | **Country of origin** |
| --- | --- | --- | --- | --- |
| 1 | JX220413.1 | BD/PPR/Netrokona-1/2011 | IV | Bangladesh |
| 2 | MG581412.1 | PPRV/Bangladesh/BD2/2008, | IV | Bangladesh |
| 3 | KX860044.1 | PPRV-UP-BRY-70/2008 | IV | India |
| 4 | KX860043.1 | PPRV-GUJ-8/2009 | IV | India |
| 5 | KX860031.1 | PPRV-RAJ-DHOL-38/2006 | IV | India |
| 6 | KX860030.1 | PPRV-RAJ-DHOL-31/2005 | IV | India |
| 7 | KX860027.1 | PPRV-UP-SHAHJ-18/2004 | IV | India |
| 8 | KX860025.1 | PPRV-WB-NAD-23/2004 | IV | India |
| 9 | KX860014.1 | PPRV-BIH-PAT-13/2012 | IV | India |
| 10 | KX860035.1 | PPRV-MP-BHO-60/2006 | IV | India |
| 11 | JX094439.1 | BD/PPR/Mymensingh/2010 | IV | Bangladesh |
| 12 | KX860028.1 | PPRV-TN-26/2004 | IV | India |
| 13 | KT860065.1 | IND/TN/ED/2015/04 | IV | India |
| 14 | EU816772.1 | China/Tibet/07-1 | IV | China |
| 15 | JX094436.1 | BD/PPR/BAU/Sheep/2009 | IV | Bangladesh |
| 16 | KX860096.1 | PPRV-TRIP-AGAR-57/2010 | IV | India |
| 17 | KX860015.1 | PPRV-PUNJ-LUD-33/2012 | IV | India |
| 18 | KX421388.1 | China/33/2007, | IV | China |
| 19 | JX217850.1 | Tibet/Bharal/2008, | IV | Tibet |
| 20 | KX033350.1 | IND/Delhi/2016/05, | IV | India |
| 21 | KR140086.1 | Izatnagar/94, | IV | India |
| 22 | KJ867542.1 | Sungri/1996/MSD | IV | India |

**Supplementary Table S2.** List of accession no., lineage and country of origin of **H gene** isolates of PPRV in GenBank with their accession number (**Accession number of our H gene - MH999830**)

| **Serial no.** | **Accession no** | **H gene isolates** | **Lineage** | **Country of origin** |
| --- | --- | --- | --- | --- |
| 1 | GQ184301.1 | x11 hemagglutinin gene | IV | China |
| 2 | KR140086.1 | Izatnagar/94 | IV | India |
| 3 | KX421388.1 | KX421388.1 | IV | China |
| 4 | JX217850.1 | Tibet/Bharal/2008 | IV | China |
| 5 | JF939201.1 | China/Tib/07 | IV | China |
| 6 | KT860065.1 | IND/TN/ED/2015/04 | IV | India |
| 7 | KX033350.1 | IND/Delhi/2016/05 | IV | India |
| 8 | KJ867541.1 | Ethiopia 2010 | IV | Ethiopia |
| 9 | MG581412.1 | PPRV/Bangladesh/BD2/2008 | IV | Bangladesh |
| 10 | KT860064.1 | IND/TN/VEL/2015/03 | IV | India |
| 11 | KU053340.1 | PPRV/Chousinga/2013 | IV | India |
| 12 | KF727981.2 | Sungri/96 | IV | India |
| 13 | KX189062.1 | Egypt/Giza-3/2015 | IV | Egypt |
| 14 | KC594074.1 | Morocco 2008 | IV | Morocco |
| 15 | FJ750563.1 | Bhopal 2003 | IV | India |
| 16 | MF737202.1 | Georgia/Tbilisi/2016 | IV | Georgia |
| 17 | KY888168.1 | PPRV/Mongolia/9/2016 | IV | Mongolia |
| 18 | KF648288.1 | Kurdistan 2011 | IV | Iraq |
| 19 | KR781450.1 | Benin/B1/1969 | II | Benin |
| 20 | MF741712.1 | PPRV/Sierra Leone/048/2011 | II | Sierra Leone |
| 21 | KY628761.1 | Morbillivirus strain 75/1 | I | Nigeria |
| 22 | KU236379.1 | Lib/2015 | II | Liberia |
| 23 | KJ466104.1 | Ghana/NK1/2010 | II | Ghana |
| 24 | HQ197753.1 | Nigeria/75/1 | I | Nigeria |
| 25 | EU267274.1 | Ng76/1 | I | Nigeria |


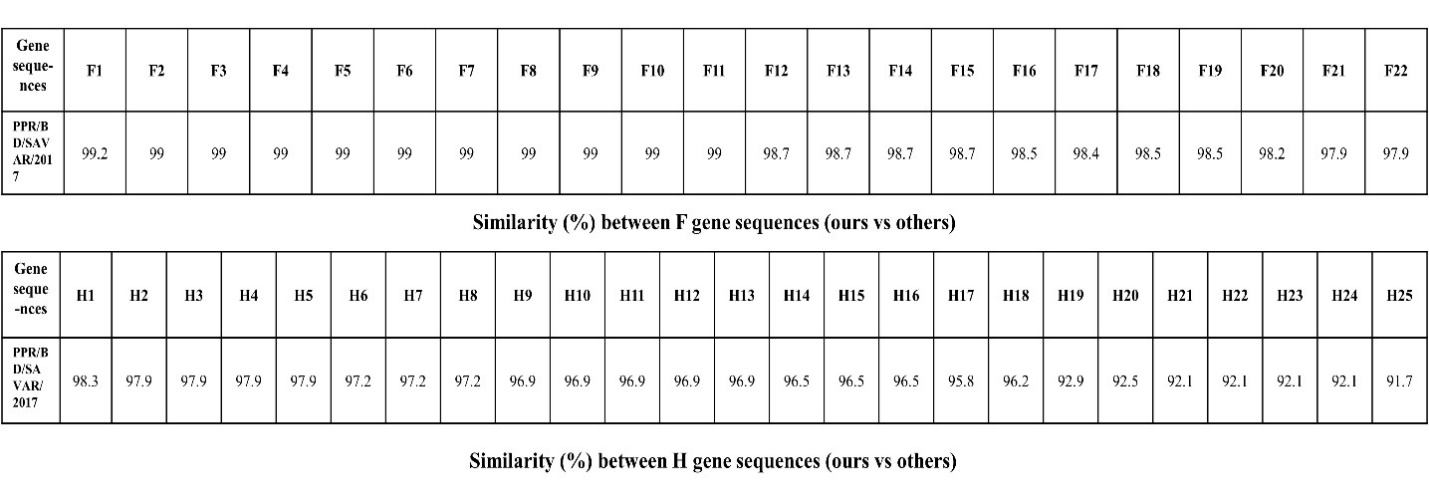


**Supplementary Figure S1.** Similarities of our F and H gene sequences with other F (F1-F22) and H (H1-H25) gene sequences of PPR virus isolates of sheep. Here, **F1**= BD/PPR/Netrokona-1/2011, **F2**= PPRV/Bangladesh/BD2/2008, **F3**= PPRV-UP-BRY-70/2008, **F4**= PPRV-GUJ-8/2009. **F5**= PPRV-RAJ-DHOL-38/2006, **F6**= PPRV-RAJ-DHOL-31/2005, **F7**= PPRV-UP-SHAHJ-18/2004, **F8**= PPRV-WB-NAD-23/2004, **F9**= PPRV-BIH-PAT-13/2012, **F10**= PPRV-MP-BHO-60/2006, **F11**= BD/PPR/Mymensingh/2010, **F12**= PPRV-TN-26/2004, **F13**= PPRV/IND/TN/ED/2015/04, **F14**= PPRV/China/Tibet/07-1, **F15**= BD/PPR/BAU/Sheep/2009, **F16**= PPRV-TRIP-AGAR-57/2010, **F17**= PPRV-PUNJ-LUD-33/2012, **F18**= Morbillivirus China/33/2007, **F19**= PPRV/Tibet/Bharal/2008, **F20**= PPRV/IND/Delhi/2016/05, **F21**= PPRV/Izatnagar/94, **F22**= PPRV/Sungri 1996 MSD, **H1**= PPRV/Tibet/2010, **H2**= PPRV/Izatnagar/94, **H3**= PPRV/China/33/2007, **H4**= PPRV/Tibet/Bharal/2008, **H5**= PPRV/China/Tib/07, **H6**= PPRV/IND/TN/ED/2015/04, **H7**= PPRV/IND/Delhi/2016/05, **H8**= PPRV/Ethiopia/2010, **H9**= PPRV/Bangladesh/BD2/2008, **H10**= PPRV/IND/TN/VEL/2015/03, **H11**= PPRV/Chousinga/2013, **H12**= PPRV/Sungri/96, **H13**= PPRV/Egypt/Giza-3/2015, **H14**= PPRV/Morocco/2008, **H15**= PPRV/Bhopal/2003, **H16**= PPRV/Georgia/Tbilisi/2016, **H17**= PPRV/Mongolia/9/2016, **H18**= PPRV/Kurdistan/2011, **H19**= PPRV/Benin/B1/1969, **H20**= PPRV/SierraLeone/048/2011, **H21**= Nigeria/75/1, **H22**= PPRV/Lib/2015, **H23**= PPRV/Ghana/NK1/2010, **H24**= PPRV/Nigeria/75/1, **H25**= PPRV/Ng76/1.
